# TRPM7 inhibitor carvacrol protects brain from neonatal hypoxic-ischemic injury

**DOI:** 10.1186/s13041-015-0102-5

**Published:** 2015-02-19

**Authors:** Wenliang Chen, Baofeng Xu, Aijiao Xiao, Ling Liu, Xiaoyan Fang, Rui Liu, Ekaterina Turlova, Andrew Barszczyk, Xiao Zhong, Christopher L F Sun, Luiz R G Britto, Zhong-Ping Feng, Hong-Shuo Sun

**Affiliations:** Department of Surgery, Faculty of Medicine, University of Toronto, 1 King’s College Circle, Toronto, M5S 1A8 Canada; Department of Physiology, Faculty of Medicine, University of Toronto, 1 King’s College Circle, Toronto, M5S 1A8 Canada; Department of Pharmacology, Faculty of Medicine, University of Toronto, 1 King’s College Circle, Toronto, M5S 1A8 Canada; Institute of Medical Science, Faculty of Medicine, University of Toronto, 1 King’s College Circle, Toronto, M5S 1A8 Canada; Faculty of Applied Science & Engineering, University of Toronto, Toronto, M5S 1A4 Canada; Instituto de Ciências Biomédicas, Universidade de São Paulo, São Paulo, Brazil

**Keywords:** TRPM7, Neonatal hypoxic-ischemic brain injury, Neuroprotection, Carvacrol

## Abstract

**Background:**

Our previous study found that suppression of TRPM7 reduced neuronal death in adult rat ischemic brain injury. It was reported that carvacrol blocked TRPM7 and attenuated brain injury in an adult rat MCAO model. The effects of carvacrol on neonatal stroke remain unknown. This study investigated the effects of carvacrol on neuronal injury and behavioral impairment after hypoxia-ischemia in neonatal mice and the potential signaling pathway underlying these effects.

**Results:**

Carvacrol inhibited TRPM7 current in HEK293 cells over-expressing TRPM7 and TRPM7-like current in hippocampal neurons in a dose-dependent manner. Carvacrol (>200 μM) reduced OGD-induced neuronal injury in cortical neurons. 24 hours after HI, TRPM7 protein level in the ipsilateral hemisphere was significantly higher than in the contralateral hemisphere. Carvacrol (30 and 50 mg/kg) pre-treatment reduced brain infarct volume 24 hours after HI in a dose-dependent manner. Carvacrol pre-treatment also improved neurobehavioral outcomes. Furthermore, animals pre-treated with carvacrol had fewer TUNEL-positive cells in the brain compared to vehicle-treated animals 3 days after HI. Carvacrol pre-treatment also increased Bcl-2/Bax and p-Akt/t-Akt protein ratios and decreased cleaved caspase-3 protein expression 24 hours after HI.

**Conclusions:**

Carvacrol pre-treatment protects against neonatal hypoxic-ischemic brain injury by reducing brain infarct volume, promoting pro-survival signaling and inhibiting pro-apoptotic signaling, as well as improving behavioral outcomes. The neuroprotective effect may be mediated by the inhibition of TRPM7 channel function. Carvacrol is a potential drug development target for the treatment of neonatal stroke.

## Background

Hypoxic-ischemic injury or stroke in mammalian brains elicits delayed neuronal death (DND) [1]. Previous studies have considered glutamate excitotoxicity as a key mechanism in stroke [2]. However, the inhibition of glutamate signaling has not been sufficiently neuroprotective in clinical trials [3]. Recently, non-glutamate mechanisms have attracted more attention in stroke research. Non-glutamate mechanisms also lead to intracellular ionic imbalance and neuronal cell death in ischemia and stroke [4]. TRPM7 is an important non-glutamate mechanism and thus a potential target for drug development against ischemic brain injury [4].

Transient receptor potential melastatin 7 (TRPM7) belongs to the melastatin-related subfamily of TRP channels and is ubiquitously expressed in almost all tissues, including brain [5]. TRPM7 is a Ca^2+^-permeable, non-selective cation channel that has recently gained attention as a potential cation influx pathway involved in ischemic neuronal injury. An earlier study has demonstrated that TRPM7 is a key mediator of anoxic neuronal death [6,7]. Subsequent reports have revealed that TRPM7 mRNA and protein expression increase in the brain after cerebral ischemic injury and in hippocampal neurons subjected to oxygen-glucose deprivation (OGD) [7-10]. Moreover, it is found that Lactuside B and Ginsenoside-Rd protect against cerebral ischemic damage, at least in part by decreasing TRPM7 expression. *In vivo* study has shown that TRPM7 suppression by virally mediated gene silencing prevents delayed neuronal cell death and promotes neurobehavioral functional recovery in a rat global cerebral ischemia model [11]. Hence, TRPM7 seems to be a promising potential therapeutic target for drug development for stroke.

Carvacrol, a pungent natural compound often used as a food additive, has been reported to block TRPM7 current in HEK cells over-expressing TRPM7 and in hippocampal neurons [12], as well as provide neuroprotection in adult mice subjected to focal ischemia [13]. Carvacrol is therefore a potential pharmacological tool for studying the functions of TRPM7 channels *in vitro* and *in vivo*.

Neonatal hypoxic-ischemic (HI) brain injury (also known as neonatal stroke) and its related brain disorders hypoxic-ischemic encephalopathy (HIE) and cerebral palsy, are a major cause of morbidity and mortality in infants and children, with a reported incidence of 2–9 per 1000 births [14]. Approximately 20–50% of infants that have suffered HI will die and up to 25% will suffer permanent brain damage [14]. It causes severe neurological disorders and lifelong disability in survivors, including cerebral palsy, mental retardation, and epilepsy [15]. Currently, there are no effective pharmacological interventions available for protecting neonatal brains after HI injury. The effect of carvacrol in neonatal stroke has not yet been studied. Here we report the neuroprotective effects of carvacrol in mouse neonatal hypoxic-ischemic brain injury.

## Results

### Carvacrol inhibits TRPM7 currents in HEK293 cells and hippocampal neurons

**Figure 1:**
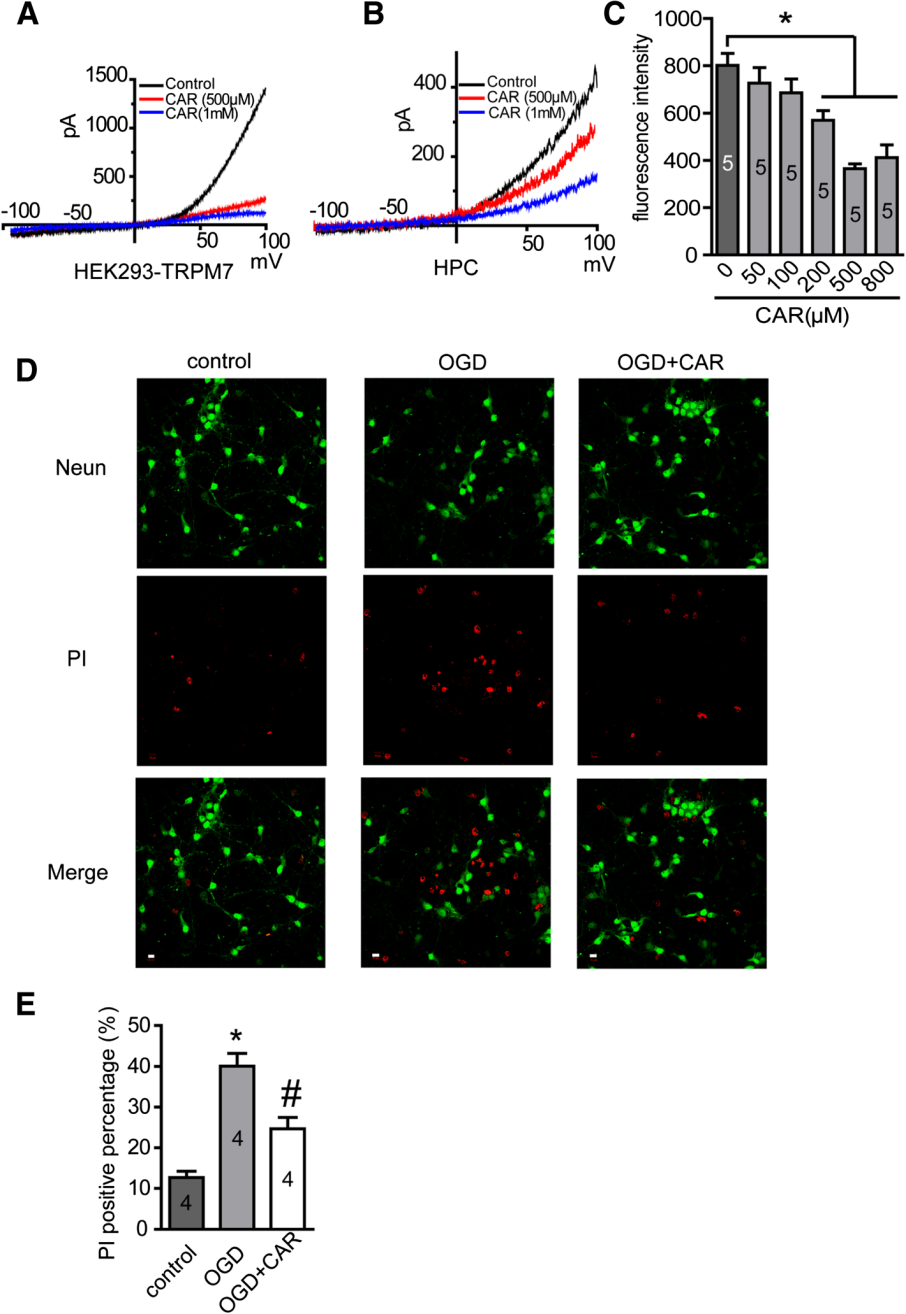
**Carvacrol (CAR) inhibits TRPM7 and TRPM7-like currents and protects cortical neurons from OGD-induced injury. A**, HEK293 cells over-expressing TRPM7 were induced by tetracycline (1 μM) for 24 hours. TRPM7 current was recorded as described in methods section. Representative I-V curves are shown. Perfusion with carvacrol (500 μM and 1 mM) caused a dramatic decrease in the TRPM7 current in dose-dependent manner (n = 6 cells). **B**, TRPM7-like current in primary hippocampal neurons (HPC) was recorded as described in methods section. Perfusion with carvacrol (500 μM and 1 mM) dose-dependently blocked TRPM7-like current in HPC. Representative I-V curves are shown (n = 6 cells). **C**, cortical neurons were incubated with carvacrol or vehicle (0.1% DMSO) for 30 min and then treated with OGD for 1 hour and transferred to regular medium for 24 hours. Cells were then stained with PI and the fluorescent intensity was measured using Synergy HT Multi-Mode Micro plate Reader. Results demonstrated that carvacrol (200-800 μM) significantly protected neurons from OGD-induced injury (*, p < 0.05 compared with vehicle treated group, n = 5, One-way ANOVA followed by Newman-Keuls test). **D** and **E**, cortical neurons were treated with carvacrol (300 μM) for 30 min, and then OGD and PI staining were conducted as described above. Representative images were taken using a Zeiss LSM 710 Confocal Microscope. Scale bar = 10 μm. *, p < 0.05 compared with control group; #, p < 0.05 compared with OGD group, n = 4, One-way ANOVA followed by Newman-Keuls test.

First, we performed whole-cell patch-clamp experiments to examine the effect of carvacrol on TRPM7 current in HEK293 cells over-expressing TRPM7 and on TRPM7-like native currents in mouse hippocampal neurons. Carvacrol inhibited TRPM7 current in HEK293 cells over-expressing TRPM7 (Figure 1A, n = 6 cells) and TRPM7-like currents in mouse primary hippocampal neurons (Figure 1B, n = 6 cells) in a dose-dependent manner. Thus, carvacrol is a valid pharmacological tool for further *in vitro* and *in vivo* studies.

### Carvacrol protects neurons from OGD-induced cell injury *in vitro*

We next tested whether carvacrol can protect neurons from anoxic insult (oxygen and glucose deprivation: OGD) *in vitro*. Propidium iodide (PI) fluorescence intensity in cortical neurons was significantly greater following OGD. We showed that carvacrol pre-incubation for 30 minutes significantly reduced PI fluorescence intensity in a dose-dependent manner (200-800 μM concentration) compared to that of vehicle-treat group. The PI fluorescence intensity was reduced by 28.74% at 200 μM, 54.43% at 500 μM and 48.59% at 800 μM carvacrol (Figure 1C, p < 0.05 for all three doses, n = 5). This result was further confirmed by double-staining with PI and neuronal nuclei (NeuN) antibody to evaluate the percentage of PI positive cells. As shown in Figure 1D and E, the percentage of PI positive cells was 12.7 ± 1.5% in the control group, and this increased significantly to 40.1 ± 3.1% in the OGD group (*, p < 0.05, n = 4), but was significantly less with 24.7 ± 2.8% in the carvacrol pre-treatment group (300 μM, #, p < 0.05, n = 4). This *in vitro* data indicates that carvacrol is able to protect cultured neurons from anoxic insult.

### Carvacrol pre-treatment attenuates infarct volume of hypoxic-ischemic injury *in vivo* in a dose-dependent manner

**Figure 2:**
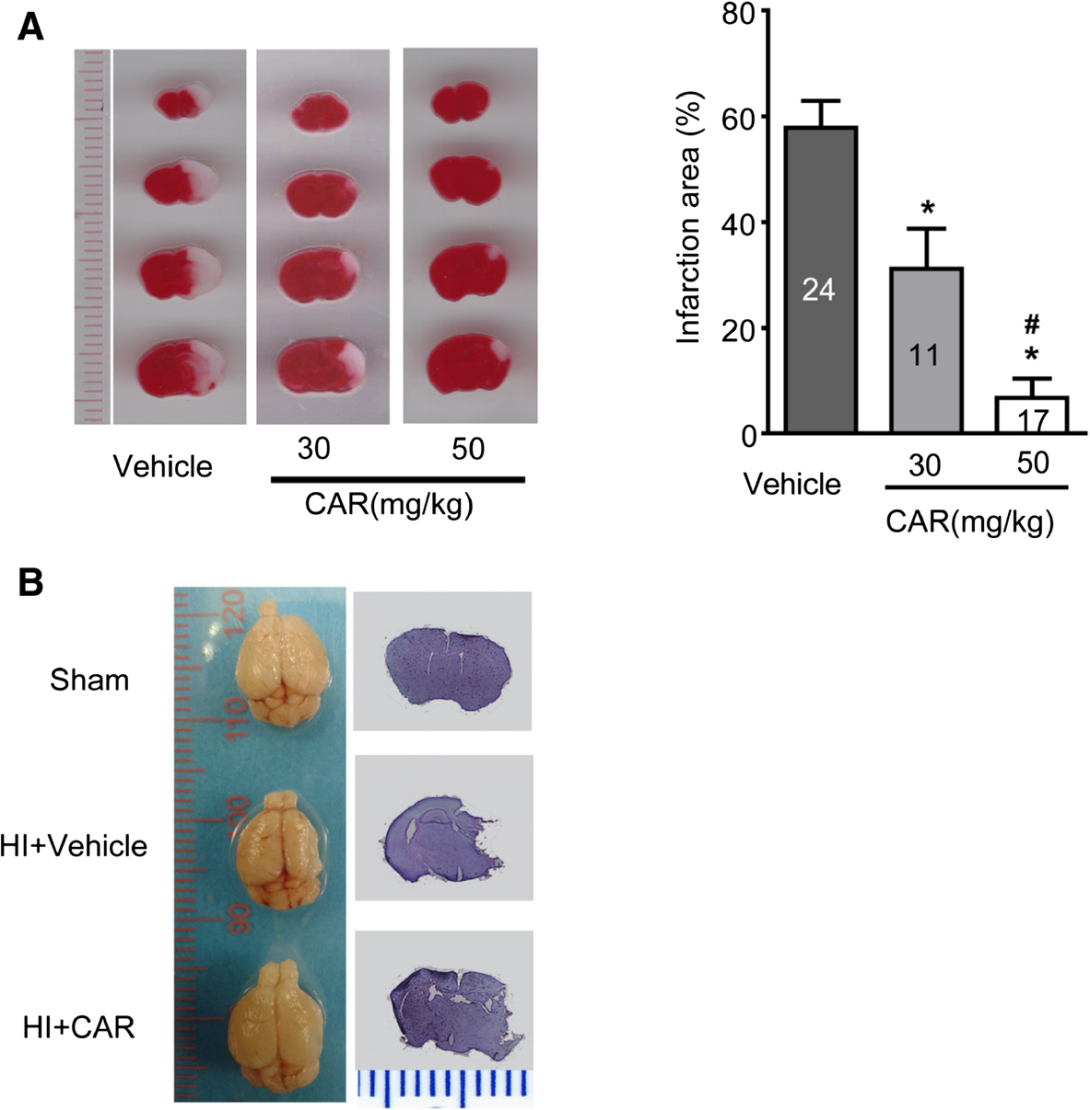
**Carvacrol (CAR) pre-treatment reduced infarct volume of neonatal hypoxic-ischemic brain injury in a dose-dependent manner. A**, TTC staining was performed as described in methods section. The representative images of TTC-stained coronal brain slices are shown (Left panel). Infarct volume is smaller in carvacrol pre-treatment group than vehicle group (*, p < 0.05 compared with vehicle group. #, p < 0.05 compared with carvacrol 30 mg/kg group, One-way ANOVA followed by Newman-Keuls test). **B**, representative images of whole brain and Nissl staining for brain slices (sham, n = 7; vehicle group, n = 12; carvacrol pretreatment group, n = 15).

Next, we asked whether carvacrol can reduce brain damage *in vivo* using a mouse neonatal hypoxic-ischemic brain injury model. We observed that carvacrol pre-treatment (30 and 50 mg/kg i.p., 30 min before HI) significantly reduced infarct volume 24 hours after HI. TTC staining of coronal sections of mouse brains was used for evaluating the infarct volume. Representative images of TTC staining were shown in Figure 2A, where white areas indicated brain damage. There was no detectable infarction in the sham group (data not shown). Infarct volume in the vehicle-treated HI group (Vehicle) was 57.83 ± 5.18% (n = 24 pups). Carvacrol pre-treatment (30 and 50 mg/kg) significantly reduced the infarct volume to 31.11 ± 7.63% (n = 11 pups) and 6.18 ± 3.73% (n = 17 pups), respectively, compared to the vehicle-treated group (*, p < 0.05). The reduction of infarct volume was dose-dependent (#, p < 0.05) (Figure 2A). Furthermore, 7 days after HI, whole brains were fixed, imaged, and then sectioned for Nissl staining. Carvacrol pre-treatment (50 mg/kg) also resulted in significantly less brain damage (both in whole brains and coronal sections, n = 15 pups) 7 days after HI (Figure 2B) compared to vehicle treatment (with larger brain tissue loss, n = 12 pups). Brain imaging at 7 days further supported TTC staining at one day with respect to the neuroprotective effects of carvacrol in HI.

### Carvacrol pre-treatment improves neurobehavioral performance after HI

**Figure 3:**
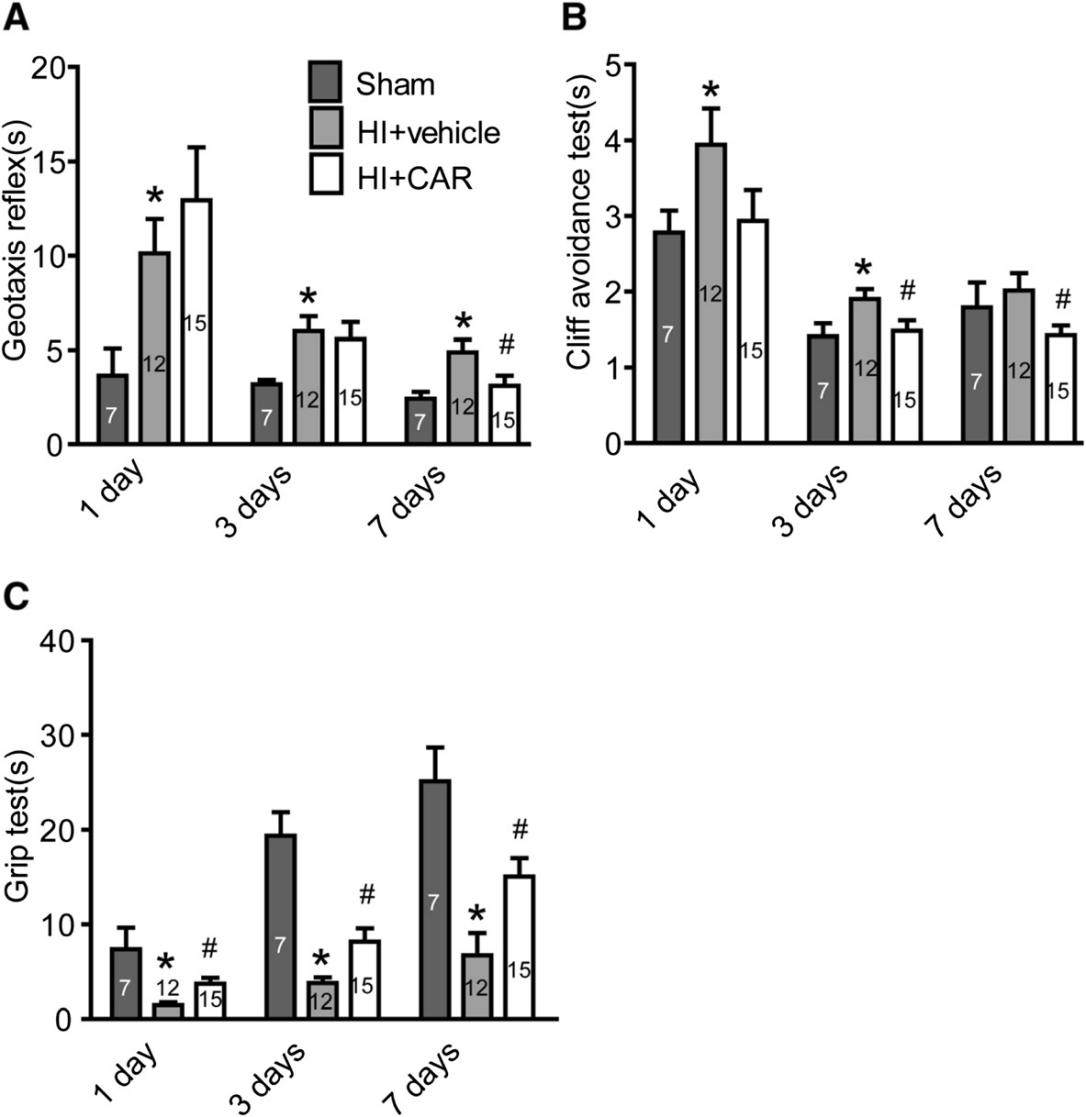
**Carvacrol pretreatment improves neurobehavioral performance after cerebral HI.** Neurobehavioral evaluation was performed as described in Material and Methods. Geotaxis reflex **(A)**, cliff avoidance test **(B)** and grip test **(C)** of sham (n = 7), vehicle group (HI + vehicle, n = 12) and carvacrol (50 mg/kg) pretreatment group (HI + CAR, n = 15) were measured 1 day, 3 days and 7 days after HI (*, p < 0.05 versus sham group; #, p < 0.05 versus vehicle group, One-way ANOVA followed by Newman-Keuls test).

We further evaluated neurobehavioral outcomes in sham (n = 7 pups), carvacrol-treated (n = 15 pups) and vehicle-treated animals (n = 12 pups). Neurobehavioral tests evaluating the geotaxis reflex, cliff avoidance and grip were performed 1, 3, and 7 days after HI in the three groups. Compared with pups in the sham group, neurobehavioral functions of pups in the vehicle-treated HI group were significantly impaired 1, 3 and 7 days after HI (Figure 3, *, p < 0.05). Geotaxis test performance was significantly better in the carvacrol-treated group 7 days after HI (Figure 3A) compared to the vehicle-treated group (3.11 ± 0.53 s in carvacrol-treated HI group versus 4.89 ± 0.67 s in the vehicle-treated HI group; p < 0.05). Cliff avoidance test performance was also significantly better in the carvacrol-treated group 3 and 7 days after HI (Figure 3B) compared to the vehicle-treated group (1.48 ± 0.14 s in the carvacrol-treated group versus 1.90 ± 0.13 s in the vehicle-treated group 3 days after HI; 1.42 ± 0.13 s in the carvacrol-treated group versus 2.01 ± 0.23 s in the vehicle-treated group 7 days after HI; p < 0.05). Grip test performance was also significantly better 1, 3 and 7 days after HI in the carvacrol-treated group compared to the vehicle-treated group (Figure 3C) (p < 0.05). Therefore, carvacrol not only reduced brain damage but also improved behavioral outcomes after HI challenge.

### TRPM7 protein expression increases in the HI mouse brain

**Figure 4:**
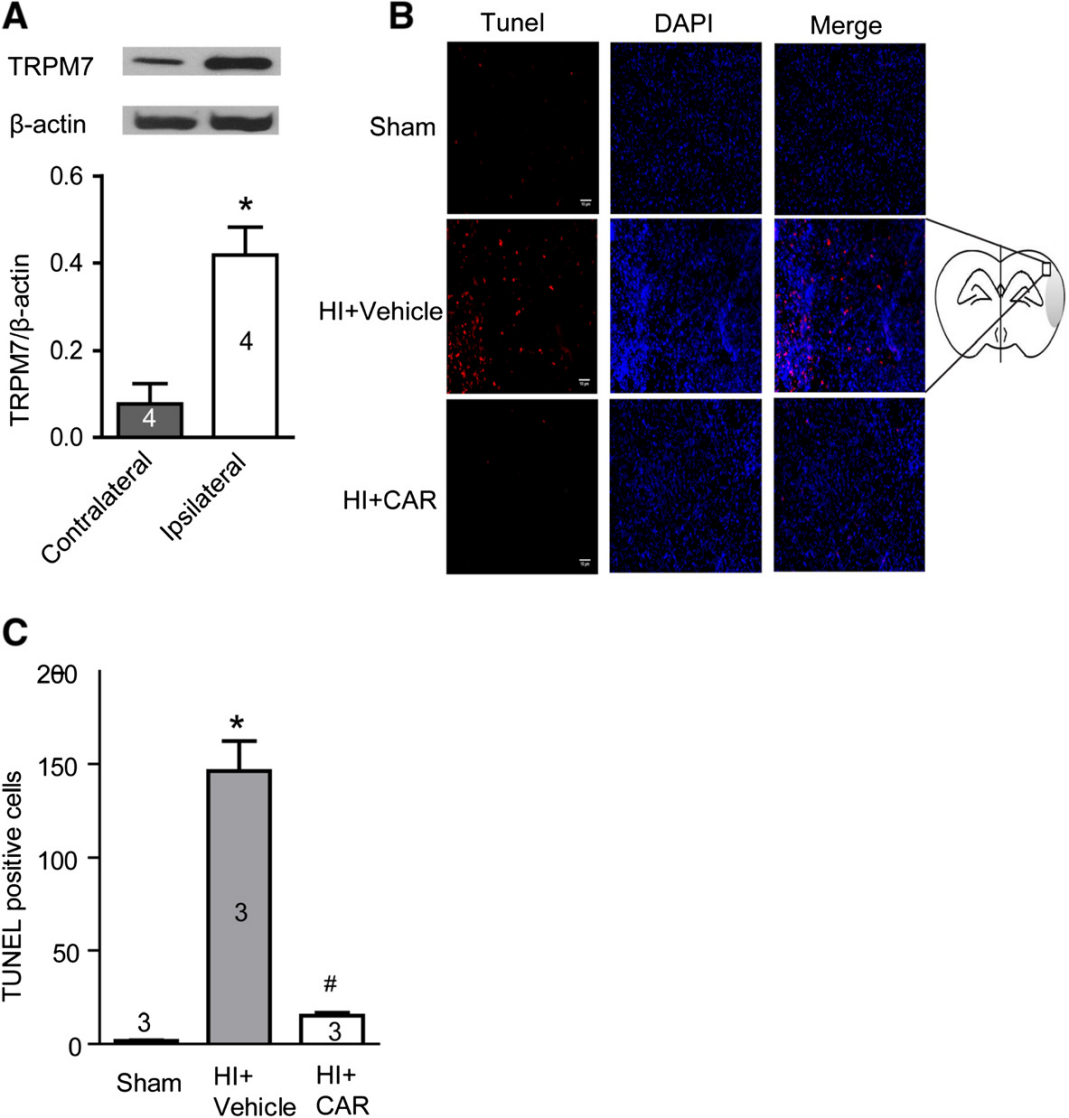
**TRPM7 protein expression in the cortex of HI mice and TUNEL staining of mouse brain slices. A**, western blotting results of TRPM7 protein expression in the injured hemisphere (ipsilateral) and uninjured contralateral hemisphere 24 hours after HI. TRPM7 protein expression level is higher in injured hemisphere than the contralateral hemisphere (*, p < 0.05, n = 4). **B**, TUNEL staining was carried out 3 days after HI and images were viewed using fluorescence microscopy. Left panel shows the representative images of TUNEL staining. Red fluorescence in TUNEL-positive cells indicates apoptosis. Blue fluorescence is DAPI staining for nucleus. Scale bar represents 10 μm. Right panel shows the mouse brain coronal sections. **C**, Quantitative analysis of TUNEL-positive cells showed that pre-treatment with carvacrol (50 mg/kg) reduced the number of TUNEL positive cells (*, p < 0.05 compared with sham group. #, p < 0.05 compared with vehicle group, n = 3, One-way ANOVA followed by Newman-Keuls test).

We also determined whether TRPM7 expression in the brain changes after HI. Western blotting experiments were carried out to compare TRPM7 protein expression between the ipsilateral and contralateral brain hemispheres 24 hours after HI. We showed that TRPM7 protein expression level was approximately 5-fold higher in the ipsilateral hemisphere than in the contralateral hemisphere (ipsilateral: 0.42 ± 0.065 arbitrary units (AUs) versus contralateral: 0.077 ± 0.047 AUs; p < 0.05, n = 4) (Figure 4A). This indicates that the TRPM7 channel may be involved in neonatal hypoxic-ischemic brain injury.

### Carvacrol pre-treatment protects brain tissue in HI model from apoptosis

**Figure 5:**
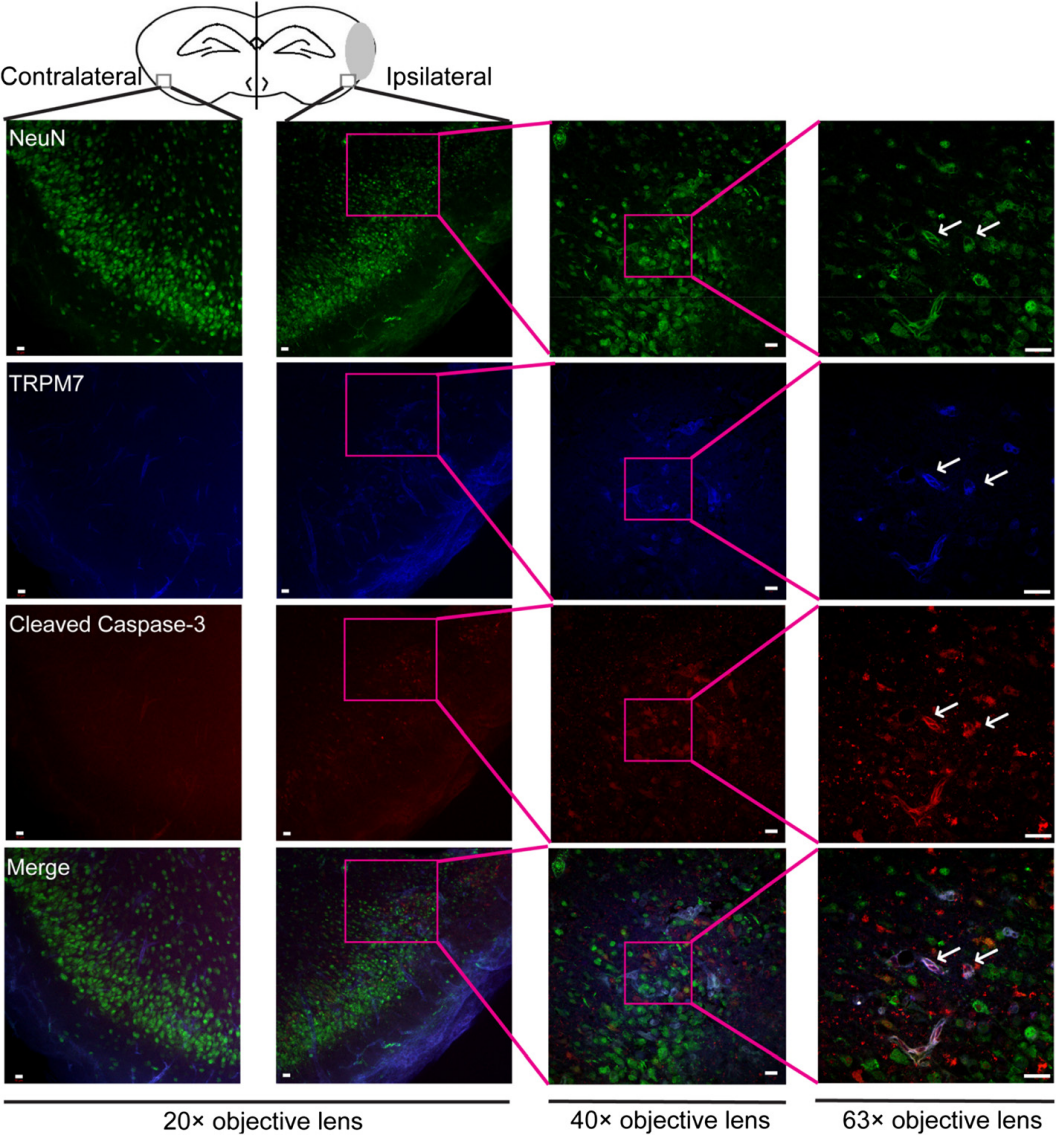
**Immunofluorescence assay of TRPM7 and cleaved caspase-3 expression in brain sections of HI mice.** Triple immunofluorescence staining was employed for TRPM7, cleaved caspase-3 and NeuN in the penumbra of the injured hemisphere (ipsilateral) and in a similar region of the uninjured contralateral hemisphere 24 hours after HI. Representative images are shown. Green, blue and red fluorescence indicates NeuN, TRPM7 and cleaved caspase-3, respectively. The white arrow indicates the co-localization of TRPM7 and activated caspase-3 with NeuN. N = 3, Scale bar in 20× and 40× magnifying images = 10 μm. Scale bar in 63× magnifying images = 20 μm.

Apoptosis is involved in neonatal HI-induced neuronal death, which may take time to develop [16,17]. It is more common in the immature brain than in the adult brain [18]. Preventing or reducing apoptosis is a therapeutic strategy for neonatal stroke. We examined whether the protective effect of carvacrol is associated with the reduction of apoptosis using a TUNEL assay and western blot (cleaved caspase-3, see below). The number of TUNEL-positive cells in the vehicle-treated HI group increased significantly with respect to the sham group (146.33 ± 16.06 cells in the vehicle-treated group versus 1.67 ± 0.33 cells in the sham group; p < 0.05, n = 3) (Figure 4B and C). Carvacrol (50 mg/kg) pre-treatment significantly decreased TUNEL-positive cell counts compared to the vehicle-treated HI group (reduced to 15.00 ± 1.46 cells, p < 0.05, n = 3). Immunofluorescence showed that over-expression of TRPM7 (blue) was accompanied by increasing expression of cleaved caspase-3 (red) in penumbra of the ipsilateral hemisphere, when compared to a similar region on the contralateral hemisphere of the HI mouse (Figure 5). Anti-NeuN antibody was used to label surviving neurons and it showed that there were more degenerating cells and fewer surviving cells in the penumbra of ipsilateral hemisphere compared with a similar region on contralateral side, which shows normal neuron morphology and mostly surviving cells. These results indicate that apoptotic cells in the penumbra of the ipsilateral hemisphere express higher levels of TRPM7 protein, and inhibition of TRPM7 by carvacrol protects cells from apoptosis in neonatal brains of HI mice. Next we investigated if this reduction of TUNEL-positive cells is mediated by any known apoptotic signals.

### Carvacrol pre-treatment increases Bcl-2/Bax protein ratio and reduces caspase-3 cleavage

**Figure 6:**
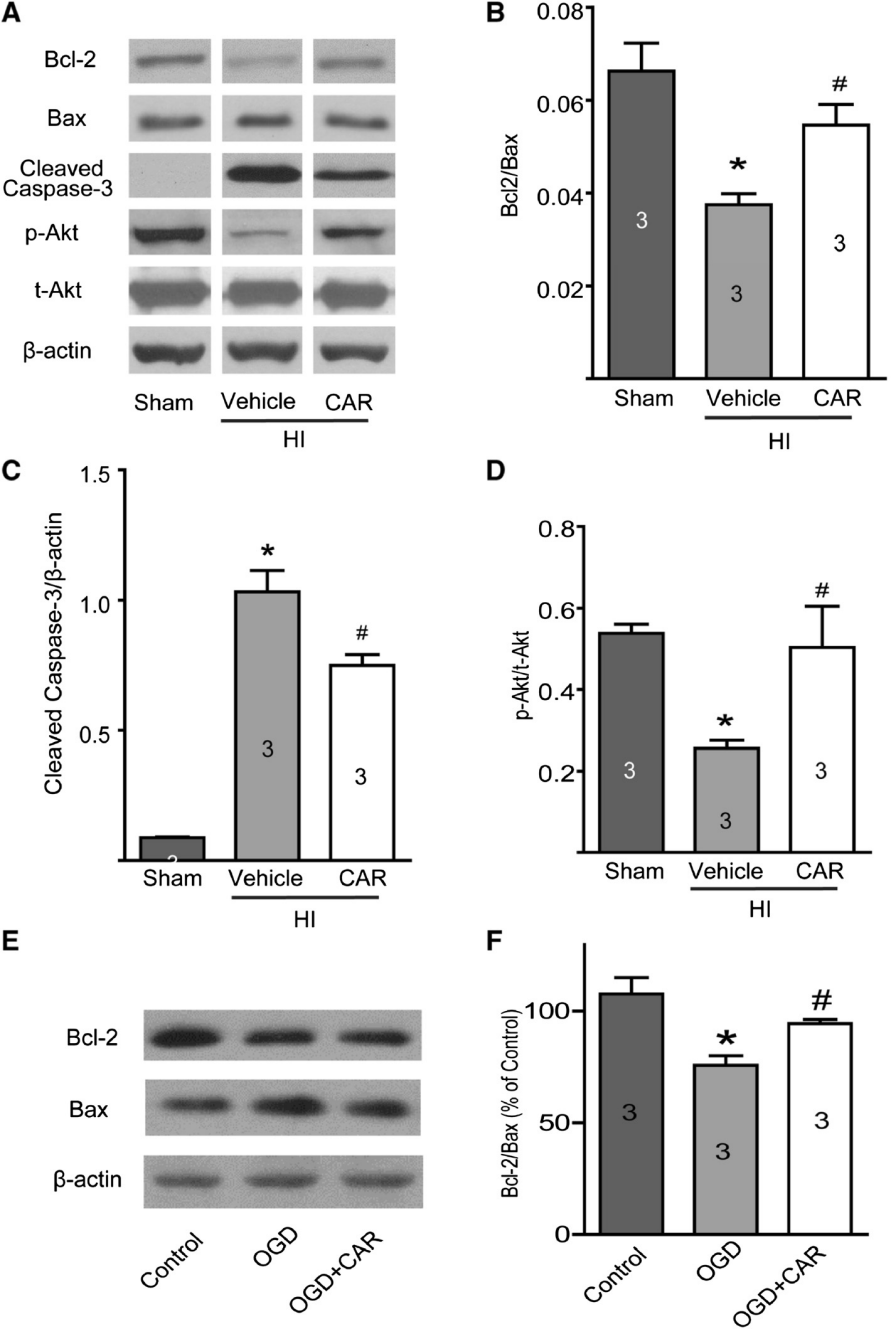
**Western blotting results. Experiments were carried out as described in the methods section. A**, representative images of Bcl-2 (26 kDa), Bax (20 kDa), cleaved caspase-3 (19/17 kDa), p-Akt (Ser 473, 60 kDa) and t-Akt (60 kDa) protein expression. β-actin (42 kDa) was used as a loading control. Carvacrol (50 mg/kg) pretreatment increased the protein ratio of Bcl-2/Bax **(B)**, decreased cleaved caspase-3 protein expression **(C)** and increased p-Akt/t-Akt protein ratio **(D)** 24 hours after HI (*, p < 0.05 versus sham group; #, *p* < 0.05 versus vehicle group, n = 3, One-way ANOVA followed by Newman-Keuls test). When primary mouse cortical neurons were exposed to OGD for 60 minutes and additional 18 hours in normal culture medium, Bcl-2/Bax ratio in the OGD group was lower than that of the control group, and significantly increased in the Carvacrol (300 μM) pre-treatment group **(E** and **F**, *, p < 0.05 versus the sham group; #, *p* < 0.05 versus the vehicle group, n = 3, One-way ANOVA followed by Newman-Keuls test).

TUNEL results indicate that carvacrol may reduce apoptosis in neonatal HI brain injury. We further determined which apoptosis-related proteins may be involved in this effect. Caspase-3 cleavage has been reported as a major cause of brain injury after neonatal stroke [19]. Bcl-2/Bax protein ratio is important in the mitochondrial apoptosis pathway [20]. Bcl-2 and Bax protein levels play a key role in brain injury in the neonatal HI model [21,22]. In accordance with other studies [13,19], we found that cleaved caspase-3 protein expression was significantly greater 24 hours after HI (normalized to β-actin, 1.03 ± 0.08 AUs in the vehicle-treated HI group versus 0.088 ± 0.003 AUs in the sham group; p < 0.05, n = 3) (Figure 6A and C). Carvacrol pre-treatment significantly reduced cleaved caspase-3 levels to 0.75 ± 0.04 AUs (p < 0.05 when compared to the vehicle-treated group, n = 3). We also found that the protein ratio of Bcl-2/Bax was significantly lower in the vehicle-treated group compared to the sham group (0.038 ± 0.002 AUs in vehicle-treated group versus 0.067 ± 0.006 AUs in sham group; p < 0.05, n = 3) (Figure 6A and B); while carvacrol pre-treatment (50 mg/kg) restored Bcl-2/Bax ratio to 0.055 ± 0.004 AUs (p < 0.05 to vehicle-treated group, n = 3). Therefore, carvacrol is able to inhibit pro-apoptotic signaling pathways in neonatal hypoxic-ischemic brain injury. Next, the effects of carvacrol on Bcl-2/Bax ratio were measured in an OGD-induced cortical neuron injury model. As shown in Figure 6E and F, cortical neurons exposed to OGD had a lower Bcl-2/Bax ratio, while the Bcl-2/Bax ratio increased in the carvacrol (300 μM) pre-treatment group (*, #, p < 0.05, n = 4).

### Carvacrol pre-treatment increases phosphorylated Akt (p-Akt) level after HI brain injury

Activation of the PI3K/Akt pathway is a protective signaling pathway involved in neuroprotection in stroke [23]. Carvacrol has been reported to increase Akt phosphorylation in a focal ischemia model [13]. In this study, we found that p-Akt/t-Akt was significantly lower in the vehicle-treated HI group (0.26 ± 0.02 AUs in the vehicle-treated group versus 0.53 ± 0.02 AUs in sham group; p < 0.05, n = 3); while carvacrol pre-treatment restored the p-Akt/t-Akt ratio to 0.50 ± 0.1 AU (p < 0.05 with respect to the vehicle-treated group, n = 3) (Figure 6A and D). Therefore, carvacrol is able to promote pro-survival signaling in neonatal hypoxic-ischemic brain injury.

## Discussion

In this study, we show that: 1) carvacrol inhibits TRPM7 and TRPM7-like currents in TRPM7 over-expressing HEK293 cells and mouse hippocampal neurons, respectively; 2) TRPM7 protein expression in the brain increases 24 hours after HI; 3) pre-treatment with the TRPM7 channel blocker carvacrol is neuroprotective and improves behavioral outcomes in a mouse model of neonatal hypoxic-ischemic brain injury; 4) carvacrol inhibits pro-apoptotic signals in HI by increasing Bcl-2/Bax protein ratio and reducing caspase-3 activation; and 5) carvacrol promotes pro-survival signals in HI by restoring the PI3K/Akt signaling pathway. These findings suggest that carvacrol has therapeutic potential in preventing and treating neonatal hypoxic-ischemic brain injury.

First, we employed whole-cell patch clamp to confirm that carvacrol inhibited TRPM7 current in HEK293 cells over-expressing TRPM7 and in cultured hippocampal neurons (Figure 1). Our results show that carvacrol inhibits TRPM7 currents in HEK293 cells and TRPM7-like currents in cultured neurons in a dose-dependent manner. This is consistent with other studies [12]. Next, we detected neuroprotective effects of carvacrol *in vitro* using an OGD model in cultured neurons. We showed that carvacrol inhibits OGD-induced neuronal cell death *in vitro* in a dose-dependent manner. As OGD is a well-accepted *in vitro* hypoxia-ischemia cell model for drug testing [24], our findings indicated that carvacrol may also protect neurons from hypoxia-ischemia brain injury *in vivo*. Hence, we further evaluated the effects of carvacrol *in vivo* using a neonatal stroke model of HI.

The neonatal hypoxic-ischemic brain injury model used in this study was adapted from the method described by Rice et al. [25], with minor modifications. It is a widely used neonatal stroke model, and has been employed to explore drug intervention targets for neonatal hypoxic-ischemic brain injury [26,27]. We have established this HI model to study the neuroprotective effects of a volume-regulated anion channel blocker [28] and hypoxic preconditioning [29]. In addition to the OGD *in vitro* model, we also evaluated the effects of carvacrol in an *in vivo* neonatal stroke model.

Upregulation of TRPM7 protein levels has been reported to occur 20 and 24 hours after MCAO and ischemia-reperfusion in adult rats [7,9]. However, brain TRPM7 protein expression after neonatal hypoxic-ischemic brain injury was previously unknown. Here we compared TRPM7 protein expression between ipsilateral and contralateral brain hemispheres from neonatal mouse pups subjected to HI 24 hours prior. Up-regulation of TRPM7 protein expression occurred in apoptotic cells in ipsilateral brain tissue. This suggests that TRPM7 is relevant to HI-induced brain injury and can potentially serve as a therapeutic target for hypoxic-ischemic brain injury, the function of which can be modulated by carvacrol.

In this study, carvacrol was intraperitoneally injected 30 minutes before pups were subjected to hypoxic-ischemic injury. Carvacrol is a lipophilic compound with a small molecular weight (mol wt, 150.22) [30]. Carvacrol likely crosses the blood–brain barrier (BBB) in neonatal mice because administering carvacrol intraperitoneally at 2 h after reperfusion showed the neuroprotective effects on brain injury in an adult mouse MCAO model [13]. The BBB of newborns is considered to be more permeable than that of adults for certain compounds [31]. Furthermore, increased BBB permeability often results from hypoxia–ischemia [32]. These factors, combined with the physicochemical characteristics of carvacrol, suggest that carvacrol crosses the BBB of HI mice. We showed that carvacrol pre-treatment reduced brain damage 1 day after HI as assessed by TTC stain and also 7 days after HI using whole brain imaging and Nissl stain. The neuroprotective effects of carvacrol are dose-dependent. Neonatal hypoxic-ischemic brain injury leads to motor and cognitive deficits, and seizures [33]. Therefore, we also evaluated the neurobehavioral function of carvacrol-treated mice compared to vehicle-treated HI and sham mice. Carvacrol improved behavioral outcomes in a battery of tests including geotaxis reflex, cliff avoidance and grip tests. Therefore, our results indicate that carvacrol not only reduced infarct volume, but also improved the return of neurobehavioral function. It was reported that carvacrol improved diabetes-related cognitive deficits in a Morris water maze test in rats [34]. The effects of carvacrol on HI-induced cognitive dysfunction remain unknown. Further experiments are required to evaluate its effects over the long term.

Apoptosis is considered to be a key pathological trigger of delayed neuronal death following transient global ischemia [16,17]. Apoptosis is more common in the immature brain than adult brain [18]. Our study detected the apoptosis in neonatal hypoxic-ischemic brain injury first using TUNEL assay followed by western blot for apoptosis-related proteins. The number of TUNEL-positive cells in the carvacrol-treated group was significantly lower than the number found in the vehicle-treated group. This is consistent with other reports showing anti-apoptotic effects of carvacrol in myocardial cells [35]. The mitochondria-dependent pathway for apoptosis is regulated by the Bcl-2 family of proteins, each of which either promotes or prevents apoptosis. Bcl-2 is involved in HI brain apoptosis [36]. The total expression of Bcl-2 decreases 24 hours after neonatal HI *in vivo* [36], while Bax expression increases. Enhancing Bcl-2 or reducing Bax expression provides protection from injury in neonatal HI brains [36-38]. The ratio of Bcl-2/Bax protein affects mitochondrial membrane permeability and regulates mitochondrially-mediated apoptosis pathways. In our study, western blotting revealed that the ratio of Bcl-2/Bax decreased in neonatal HI pups. With carvacrol pre-treatment, the protein ratio of Bcl-2/Bax was greater. In addition, carvacrol also inhibited an OGD-induced decrease in Bcl-2/Bax, suggesting that Bcl-2/Bax ratio in the mitochondrial apoptotic signaling pathway is involved in the anti-apoptotic effects of carvacrol. It is reported that Bcl-2 reduction in neonatal HI can be prevented by the calpain inhibitor CX295 [36]. M-calpain is an enzyme substrate of TRPM7 [39]. It suggests that carvacrol blocks TRPM7 function partly by reducing its enzyme substrate, m-calpain, to restore Bcl-2 expression leading to prevention of apoptosis in neonatal hypoxic-ischemic brain injury. Further research is required to reveal this mechanism. Caspase-3, which is the protein downstream of mitochondrial and death receptors in the apoptotic signaling pathway, has been identified as a key mediator of apoptosis in animal models of ischemic stroke [40]. Inhibition of caspase-3 activation is an established therapeutic strategy for neonatal HI brain injury [41,42]. In an adult MCAO mouse model, it was reported that carvacrol treatment decreased cleaved caspase-3 protein level after cerebral ischemia injury [13]. We also found that cleaved caspase-3 increased in HI pups 24 hours after hypoxia-ischemia. Carvacrol pre-treatment significantly attenuated the level of cleaved caspase-3. Hence, carvacrol effectively prevents apoptosis in neonatal hypoxic-ischemic brain injury, likely through the mitochondrial Bcl-2/Bax/caspase-3 signaling pathway.

Activation of PI3K/Akt, a critical pro-survival signaling pathway, plays a protective role in cerebral ischemia injury [23]. It has been reported that the phosphorylation level of Akt (p-Akt) is reduced 8 and 24 hours after HI in rats [43]. In our study, p-Akt protein expression was reduced 24 hours after HI, which is consistent with previous results [43]. Carvacrol pre-treatment significantly restored p-Akt protein expression. Thus, the PI3K/Akt signaling pathway also contributes to the protective effects of carvacrol in neonatal stroke.

This study’s use of a pre-treatment paradigm provides “Proof of Principle” for: 1) the involvement of a non-glutamatergic mechanism (TRPM7) in HI brain injury, and 2) the idea that TRPM7 blockers (i.e. carvacrol) are a promising target for drug development against HI-induced brain injury. Clinically, acute ischemic stroke patients are given therapies after symptom onset. It has been reported that post-stroke treatment with carvacrol also inhibited brain injury in an adult mouse model of middle cerebral artery occlusion model, with a therapeutic window of up to 6 hours [13], suggesting that carvacrol has potential as a stroke treatment. Future study will test different time courses- including post-treatment- for carvacrol in the neonatal mouse HI model and evaluate its therapeutic widow for neonatal stroke. In conclusion, carvacrol pre-treatment protects brains from hypoxic-ischemic injury, improves neurobehavioral outcomes, promotes pro-survival and inhibits pro-apoptosis signaling pathways in a mouse model of neonatal hypoxic-ischemic brain injury. Neuroprotection is likely mediated by the inhibition of TRPM7 current and the resultant up-regulation of Bcl-2/Bax and p-Akt to decrease caspase-3 activation. Since carvacrol is considered a safe substance and is widely used as a food additive [44,45], it can potentially be used as a bioactive molecule for the prevention and treatment of neonatal hypoxic-ischemic brain injury and its related brain disorders.

## Materials and methods

### Animals

All protocols were carried out in accordance with Canadian Council on Animal Care guidelines and approved by the local Animal Care Committee (Office of Research Ethics, University of Toronto). Timed-pregnant CD1 mice were purchased from Charles River Laboratories (Sherbrooke, Quebec, Canada) and gave birth in the animal facility at the University of Toronto. Mice were housed with an ambient temperature of 20 ± 1°C and a 12-hour light/dark cycle with free access to a standard laboratory chow diet and water. All experiments were performed in a blinded manner; experimenters were not aware of treatment information for all assessments.

### Materials and drugs

Carvacrol (cat#W224502), Dimethyl sulfoxide (DMSO), Tetrodotoxin (TTX), DL-2-Amino-5-phosphonopentanoic acid (APV), CNQX, nimodipine, poly-D-lysine (Sigma) and tetracycline were purchased from Sigma-Aldrich, USA. MEM medium, fetal bovine serum (FBS) and other cell culture materials were purchased from Gibco Life Technologies Corporation (USA). All other reagents used were purchased from Sigma-Aldrich, USA unless stated otherwise.

### Cell cultures

HEK293 cells with stable expression of tetracycline-inducible flag-murine TRPM7/pCDNA4 were cultured with Minimum Essential Medium (MEM) supplemented with 10% FBS, glutamax-1 (2 mM, Invitrogen, USA), blasticidin (5 μg/mL) and zeocin (0.4 mg/mL, Invitrogen, USA).

Primary neuronal culture was performed using E16 CD1 mice as described previously [11]. In brief, hippocampi or cortices were dissected and digested with 0.025% Trypsin/EDTA at 37°C for 15 min. Cell density was calculated using an Improved Neubauer hemocytometer, and then plated on poly-D-lysine-coated glass 12 mm round coverslips (German Glass, Bellco cat #1943-10012) for electrophysiology experiments, or a poly-D-lysine-coated 96-well plate for OGD experiments. The cells were kept in 5% CO_2_ and 95% humidified air at 37°C in serum-free culture medium (Neurobasal medium supplemented with 1.8% B-27, 2% HEPES, 0.25% Glutamax, and 1% antibiotic-antimyocotic).

### Electrophysiology recordings

TRPM7 and TRPM7-like currents were recorded by whole-cell patch-clamp using 400 ms voltage ramps from −100 to +100 mV, with an interval of 5 s at 2 kHz and digitized at 5 kHz using MultiClamp 700B, Digidata 1322A and Clampex 9.2 software. The pipette solution for hippocampal neurons contained (in mM): 140 CsF, 35 CsOH, 10 HEPES, 2 tetraethylammonium chloride, 11 EGTA, 1 CaCl_2_, 2 MgCl_2_ and 2 K_2_ATP (pH 7.3, 290–300 mOsm/L) [46]. The pipette solution for recordings in HEK293 cells contained (in mM): 145 cesium methanesulfonate, 10 EGTA, 8 NaCl and 10 HEPES, pH adjusted to 7.2 with CsOH. The extracellular solution for whole-cell patch-clamp recordings contained 140 mm NaCl, 5 mm KCl, 2 mm CaCl_2_, 20 mm HEPES, and 10 mm glucose, pH adjusted to 7.4 (NaOH) [46]. Patch pipette resistance was between 3–5 megaohms. When recording the TRPM7-like currents in hippocampal neurons, 500nM TTX, 25 μM APV, 40 μM CNQX, and 5 μM nimodipine was added to the bath solution.

### OGD experiment

Oxygen and glucose deprivation (OGD) has been well established in our lab [28]. In brief, cortical neurons cultured in 96-well clear plates were incubated with carvacrol in oxygen-deprived solution for 30 minutes, followed by incubation in an anaerobic chamber flushed with 5% CO_2_ and 95% N_2_ (v/v) at 37°C for 60 minutes. Cells were then incubated in normoxic conditions for an additional 24 hours.

### PI staining

OGD-induced cell injury was determined by propidium iodide (PI) staining using a microplate reader (Syngery H1, Biotek, USA) to detect fluorescence intensity. In brief, cells were incubated with PI (5 μg/ml, Molecular Probes, USA) for 20 minutes. Fluorescence intensity was read using an excitation wavelength of 488 nm, and an emission wavelength of 630 nm. PI enters the cell and stains DNA in the nucleus when cell membrane integrity is compromised during OGD. Greater fluorescence intensity indicates more cell damage in OGD. Moreover, images of PI staining were taken to show damaged cells. In brief, cells were stained with PI for 20 min, and then fixed with 4% paraformaldehyde in PBS for 20 min at RT. After washing with PBS 3 times, cells were incubated overnight with anti-NeuN antibody (1:200, Millipore, MAB377) and subsequently incubated with anti-mouse secondary antibody conjugated to Alexa Fluor® 488 (1:500, CST 4408) for 2 h at RT. Images were taken using Zeiss LSM 710 Confocal Microscope. The number of cells was counted using Image J. The percentage of PI positive cells (%) = (PI positive cell number/total cell number)*100%.

### Hypoxia–Ischemia and carvacrol administration

Mouse HI was carried out according to a method described previously [28,29]. Briefly, postnatal Day 7 (P7) mice of both genders were anesthetized with isoflurane (3.0% for induction and 1.5% for maintenance). The right common carotid artery (CCA) was exposed and ligated. Mice were then returned to their dam and allowed to recover for 1.5 hours and placed in an airtight, transparent chamber (A-Chamber A-15274 with ProOx 110 Oxygen Controller/E-720 Sensor, Biospherix, NY, USA) perfused with humidified gas mixture containing 7.5% oxygen balanced with 92.5% nitrogen at 37°C for 100 minutes. Chamber temperature was monitored using a homoeothermic blanket control unit (K-017484 Harvard Apparatus, Massachusetts, USA). Sham control animals underwent anesthesia and the common carotid artery was exposed without ligation and hypoxia.

Pups were randomly assigned to one of the following groups: sham control group (Sham), HI + vehicle (Vehicle, 0.5% DMSO in normal saline) or HI + carvacrol (CAR, 30 and 50 mg/Kg). Carvacrol was dissolved in DMSO and then diluted with normal saline (NS). Vehicle solution was prepared with equivalent DMSO in NS without carvacrol. Equivalent vehicle and carvacrol solution were administered intraperitoneally 30 min before induction of HI.

### Infarct volume evaluation, whole brain imaging and histology

2, 3, 5-Triphenyltetrazolium chloride (TTC) staining was carried out as described previously [28,29]. Briefly, 24 hours after HI, mice were anesthetized with isoflurane and sacrificed. Brains were removed and cut into four coronal sections (approximately 2 mm apart). Brain slices were immersed in TTC solution (1% in PBS, protected from light), incubated at 37°C for 15 minutes, and scanned with a high-resolution scanner (Canon). The total infarct area of each section was determined by the change in coloration. Infarct size was determined using an image-analysis system (NIH Image J). The following values were measured for each level brain slice: infarction area (A), the area of the ipsilateral hemisphere (B), and the area of the contralateral hemisphere (C). Corrected infarct volume (CIV), (%) = [(B1 + B2 + B3 + B4)-(A1 + A2 + A3 + A4)] /(C1 + C2 + C3 + C4)*100 [29,47].

Whole brains were dissected 7 days after HI and fixed in 4% paraformaldehyde overnight. Brain tissues also were sectioned coronally for Nissl staining (0.1% cresyl violet). Briefly, brain slices of 30 μm were stained with 0.1% cresyl violet for 2 minutes and rinsed quickly in distilled water. Representative images were captured using a camera (Olympus, Japan) in the same field.

### Neurobehavioral evaluation

All neurobehavioral evaluations were performed in a blinded manner. 1, 3 and 7 days after HI, animals were evaluated with 3 neurobehavioral tests [29].

Negative geotaxis is an automatic, stimulus-bound orientation movement, which is considered to be diagnostic of vestibular and/or proprioceptive function. Pups were placed head down in the middle of a plane inclined at an angle of 45° [48]. The time taken to rotate 180° was recorded. The latency to make a 180° turn was recorded up to a maximum time of 60 s.

Cliff avoidance reaction is used to assess the integration of exteroceptive input and locomotor output. Pups were placed on the edge of a platform, and it was determined how much time was required for the pup to remove both paws from the edge, either by backing off or turning away from the cliff. If the pup did not respond within 60 s, it was recorded as 60 s [49].

A grip test was used to assess force and fatigability. Pups were suspended by their forepaws on a wire stretched horizontally 30 cm over a cotton pad in a cage. The time it took for the animal to fall was recorded [50].

### Immunofluorescence staining

Brain tissues were fixed in 4% paraformaldehyde overnight and then sectioned (35 μm) using a Vibratome Series 1000. TUNEL labeling was carried out using TUNEL Apoptosis Detection Kit according to the manufacturer’s instructions (Millipore, USA, S7165) [29]. Nuclei were counterstained with DAPI. Images were taken using a Zeiss Axioscope microscope. For NeuN, cleaved caspase-3 and TRPM7 triple staining, brain slices were incubated with anti-NeuN (1:200, Millipore, MAB377), anti-cleaved caspase-3 (1:400, Cell Signaling Technology, #9661) and anti-TRPM7 (1:50, Abcam, ab729) at 4°C overnight. Then brain slices were incubated corresponding secondary antibody from Abcam (1:500): Donkey Anti-Mouse IgG H&L (Alexa Fluor® 488), Goat Anti-Rabbit IgG H&L (Alexa Fluor® 568) and Donkey Anti-Goat IgG H&L (Alexa Fluor® 405). Images were taken using Zeiss LSM 710 Confocal Microscope.

### Western blotting

Western blotting experiments were carried out as described previously [51,52]. Pups were sacrificed and brain samples were collected on dry ice 24 hours after HI. Proteins of the ipsilateral hemisphere were extracted by homogenization in RIPA buffer with proteinase inhibitor cocktail (Santa Cruz Biotechnology, USA). Protein concentrations were determined using the bicinchoninic acid (BCA) method (Pierce). Samples were separated on a 10% SDS-PAGE gel and proteins were then transferred to a nitrocellulose membrane (200 mA per gel, 60 min). Blots were probed with anti-TRPM7 antibody (1:1000, Abcam, ab85016), anti-Bax Antibody (1:1000), anti-Bcl-2 (D17C4) Rabbit mAb (1:1000), anti-phospho-Akt (Ser473, 1:1000), anti-Akt (C67E7, 1:1000) and anti-cleaved caspase-3 (1:500) antibodies (above antibodies were purchased from Cell Signaling Technology, Danvers, Mass). Horseradish peroxidase–conjugated anti-mouse or anti-rabbit IgG antibodies (1:7500, Sigma) were used as secondary antibodies and were detected with the ECL system (PerkinElmer, Inc, USA). β-actin (1:3000, Sigma) was used as a control for protein loading. Images were analyzed using an image-analysis system (NIH Image J 1.47v). Protein expression was normalized to that of β-actin and expressed as percentage of control.

## Data analysis

Data is presented as mean ± SEM. Statistical differences between groups were analyzed using a one-way ANOVA with subsequent Newman-Keuls test for multiple comparisons and Student’s *t*-test to compare two groups. p < 0.05 was considered statistically significant.
